# Modeling of Hydration, Compressive Strength, and Carbonation of Portland-Limestone Cement (PLC) Concrete

**DOI:** 10.3390/ma10020115

**Published:** 2017-01-26

**Authors:** Xiao-Yong Wang

**Affiliations:** Department of Architectural Engineering, College of Engineering, Kangwon National University, Chuncheon 200701, Korea; wxbrave@kangwon.ac.kr; Tel.: +82-33-250-6229

**Keywords:** Portland-limestone cement concrete, hydration, compressive strength, carbonation, model

## Abstract

Limestone is widely used in the construction industry to produce Portland limestone cement (PLC) concrete. Systematic evaluations of hydration kinetics, compressive strength development, and carbonation resistance are crucial for the rational use of limestone. This study presents a hydration-based model for evaluating the influences of limestone on the strength and carbonation of concrete. First, the hydration model analyzes the dilution effect and the nucleation effect of limestone during the hydration of cement. The degree of cement hydration is calculated by considering concrete mixing proportions, binder properties, and curing conditions. Second, by using the gel–space ratio, the compressive strength of PLC concrete is evaluated. The interactions among water-to-binder ratio, limestone replacement ratio, and strength development are highlighted. Third, the carbonate material contents and porosity are calculated from the hydration model and are used as input parameters for the carbonation model. By considering concrete microstructures and environmental conditions, the carbon dioxide diffusivity and carbonation depth of PLC concrete are evaluated. The proposed model has been determined to be valid for concrete with various water-to-binder ratios, limestone contents, and curing periods.

## 1. Introduction

Portland-limestone cement (PLC) is manufactured by intergrinding Portland cement clinker with various contents of limestone. The limestone is more easily ground than clinker, and becomes concentrated in the finest particles. Many benefits can be achieved by using PLC concrete. PLC concrete shows better workability and less bleeding than control concrete. When the replacement ratio of limestone is less than 5%, the performance of concrete is not affected. In addition, ecological advantages, such as reductions in CO_2_ and NO*_x_* emissions from cement manufacturing, can be obtained by using PLC concrete [1,2].

On the other hand, besides the normal cement content (around 300 kg/m^3^ of concrete), limestone is added as filler material in quantities of 200 to 300 kg/m^3^ for producing self-consolidating concrete. This study focuses on PLC concrete rather than limestone-blended self-consolidating concrete. In this study, water-to-binder ratio (W/B) means the mass ratio of water to Portland cement plus limestone, and water-to-cement ratio (W/C) means the mass ratio of water to Portland cement.

Hydration, compressive strength development, and carbonation resistance are crucial factors for the practical use of PLC concrete. Many experimental studies have been performed and many theoretical models have been developed for studying PLC concrete. Bonavetti et al. [3] found that limestone replacement (replacing a portion of the Portland cement with limestone) could increase the water-to-cement (W/C) ratio and the degree of hydration of cement. Therefore, the limestone used in low W/B ratio concrete is considered a rational use of energy. Elgalhud et al. [4] reported that the properties related to the pore structure of concrete remained unimpaired up to a 25% maximum replacement of limestone-to-binder materials. Beyond 25% replacement, the pore structure begins to deteriorate. Bentz et al. [5] found that the early-age strength of PLC concrete is higher than that of control concrete. Parrott [6] and Balayssac [7] et al. found that limestone replacements increase the carbonation depth of concrete. With the extension of curing periods, the carbonation resistance of concrete increases.

Aside from experimental studies [3,4,5,6,7], abundant numerical models have been proposed to predict properties of PLC concrete. Kishi and Saruul [8] and Maekawa et al. [9] proposed a model to evaluate the heat evolution rate of PLC. The effects of limestone on the reaction-controlling stage and the diffusion-controlling stage in cement hydration were considered. Poppe et al. [10] and Ye et al. [11] simulated the hydration process and microstructure development of PLC concrete. The heat evolution rate and porosity were calculated using the degree of hydration. Cyr et al. [12] proposed an efficiency function to consider the effect of limestone on the strength development of concrete. This efficiency function [12] was also adopted by Zeng et al. [13] to simulate the heterogeneous nucleation effect of fly ash on cement hydration. Bentz [14,15] simulated the hydration of PLC. The dilution effect, physical effect (nucleation effect), and chemical effect (formation of monocarboaluminate phase) were modeled. Lothenbach et al. [16] created thermodynamic modeling of PLC. The evolution of phase volume fractions of hydration products were calculated. In summary, current hydration models mainly focus on cement-limestone hydration [8,9,10,11,16] and strength development [12,13,14,15]. The relations between cement-limestone hydration and durability aspects, such as carbonation, are scarcely considered.

Carbonation is one of the main causes of corrosion initiation in steel rebar in reinforced-concrete (RC) structures [9]. The service life of RC structures in an atmospheric environment is closely related to carbonation. Analytical models were proposed to evaluate the carbonation resistance of concrete. Demis et al. [17] calculated the contents of carbonatable materials and concrete porosity. The carbonation depth of concrete was predicted by considering concrete material properties and environmental conditions. Kwon and Na [18] and Ann et al. [19] considered the uncertainties involved with carbonation prediction and proposed a probabilistic approach to evaluate the carbonation depth of concrete. However, Demis et al. [17] assumed that all of the binders in concrete would hydrate regardless of water-to-binder ratio. Maekawa et al. [9] reported that concrete with a lower W/B has a slower hydration rate and a lower ultimate degree of hydration. In addition, Marques et al. [20,21] found that with an extension in the curing period, the carbonation resistance of concrete was enhanced. Current carbonation models [17,18,19] do not consider the effect of curing periods on carbonation.

To overcome the shortcomings in former studies [3,4,5,6,7,8,9,10,11,12,13,14,15,16,17,18,19], this study presents a numerical model to systematically evaluate the hydration kinetics, compressive strength development, and carbonation depth of PLC concrete. By using a PLC hydration model, the hydration degree of cement, the amount of reaction products, porosity, gel–space ratio, and compressive strength are predicted. Furthermore, the calculation results from the hydration model are used as input parameters for the carbonation model. In addition, CO_2_ diffusivity and the carbonation depth of PLC concrete are evaluated, considering material properties and environmental conditions.

## 2. Hydration Model

### 2.1. Hydration Model for Cement

Wang and Lee [22] proposed an enhanced shrinking-core model to simulate Portland cement hydration. The shrinking core model considers the influences of the W/C ratio, cement compound compositions, and capillary water contents on cement hydration. The hydration model analyzes the kinetic processes during cement hydration, such as initial dormant period, the activated chemical reaction controlled process, and the diffusion controlled process. The equation for the hydration model is shown below:

(1) $$\frac{d\alpha }{dt}=\frac{3\left(S_{w}/S_{0}\right)\rho_{w}C_{w-free}}{(v+w_{g})r_{0}\rho_{c}}\frac{1}{(\frac{1}{k_{d}}-\frac{r_{0}}{D_{e}})+\frac{r_{0}}{D_{e}}{\left(1-\alpha \right)}^{\frac{-1}{3}}+\frac{1}{k_{r}}{\left(1-\alpha \right)}^{\frac{-2}{3}}}$$

where α represents the reaction degree of cement, $k_{d}$ is the reaction coefficient in the initial dormant period, $D_{e}$ is the reaction coefficient in the diffusion-controlled stage, $k_{r}$ is the reaction coefficient of the boundary reaction process, ν denotes the stoichiometric ratio by mass of water to mass of cement (=0.25), $w_{g}$ denotes the physically bound water in hydration products (=0.15; the values of ν and $w_{g}$ depend on the compound compositions of cement [9]. In this study, for simplicity, fixed values for ν and $w_{g}$ are used), $\rho_{w}$ denotes the density of water, $\rho_{c}$ denotes the density of the cement, $C_{w-free}$ denotes the amount of capillary water at the exterior of hydration products, $r_{0}$ denotes the radius of unhydrated cement particles ($r_{0}=\frac{3}{\rho_{C}S_{C}}$, where $S_{C}$ is the Blaine surface area of cement), $S_{w}$ denotes the effective contacting surface area between the cement particles and capillary water, and $S_{0}$ denotes the total surface area if hydration products develop unconstrained.

The initial dormant period consists of the formation of an initial impermeable layer and the destruction of this impermeable layer. The rate of hydration decreases because of the formation of this impermeable layer. Conversely, the rate of hydration increases when this impermeable layer is destroyed. The reaction coefficient, $k_{d}$, can be determined as follows:

(2) $$k_{d}=\frac{B}{\alpha^{1.5}}+C\alpha^{3}$$

where *B* is the rate of the initial impermeable layer formation, and *C* is the rate of the initial impermeable layer decay.

The parameter, $D_{e}$, represents the rate of cement hydration in the diffusion-controlled stage. $D_{e}$ can be calculated as a function of the degree of hydration, as follows:

(3) $$D_{e}=D_{e0}\ln(\frac{1}{\alpha })$$

where $D_{e0}$ is the initial diffusion coefficient.

The amount of water in the capillary pores, $C_{w-free}$, is determined as a function of hydration degree as shown in Equation (4):

(4) $$C_{w-free}={\left(\frac{W_{0}-0.4\times \alpha \times C_{0}}{W_{0}}\right)}^{r}$$

where $C_{0}$ is the cement content in mixing proportion, $W_{0}$ is the water content in the mix proportion, *r* is an empirical parameter that considers the accessibility of water into an inner anhydrous part through an outer hard shell of cement particles. When the W/C ratio is higher than 0.4, r=1.0; and when W/C is less than 0.4, because of the increasing constrictivity and tortuosity in capillary pores and decreasing of pore connectivity of, r is higher than 1, and r can be determined as $r=2.6-4\frac{W_{0}}{C_{0}}$ [22]. For high-strength concrete with low W/C ratio at late ages, $C_{w-free}$ has significant influence on the rate of hydration.

The influences of temperature on reaction coefficients can be described by using Arrhenius’s law [22] as follows:

(5) $$B=B_{20}\exp(-\beta_{1}(\frac{1}{T}-\frac{1}{293}))$$

(6) $$C=C_{20}\exp(-\beta_{2}(\frac{1}{T}-\frac{1}{293}))$$

(7) $$k_{r}=k_{r20}\exp(-\frac{E}{R}(\frac{1}{T}-\frac{1}{293}))$$

(8) $$D_{e0}=D_{e20}\exp(-\beta_{3}(\frac{1}{T}-\frac{1}{293}))$$

where $\beta_{1}$, $\beta_{2}$, E/R, and $\beta_{3}$ denote temperature sensitivity coefficients, and $B_{20}$, $C_{20}$, $k_{r20}$, and $D_{e20}$ represent the values of B, C, $k_{r}$, and $D_{e}$, respectively, at 20 °C.

Wang [23] studied the dependence of the reaction coefficients of the hydration model in cement compound compositions. Five types of Portland cement were used: ordinary Portland cement, early-hardening cement, moderate-heat cement, low-heat cement, and belite-rich cement. The relationship between the hydration reaction coefficients and the cement compound compositions was deduced based on the analysis of the degree of hydration and the adiabatic temperature rise of hardening concrete. These relationships are shown as follows:

(9) $$B_{20}=6\times 10^{-12}\times (C_{3}S\%+C_{3}A\%)+4\times 10^{-10}$$

(10) $$C_{20}=0.0003\times C_{3}S\%+0.0186$$

(11) $$kr_{20}=8\times 10^{-8}\times C_{3}S\%+1\times 10^{-6}$$

(12) $$De_{20}=-8\times 10^{-12}\times C_{2}S\%+7\times 10^{-10}$$

(13) $$\beta_{1}=1000$$

(14) $$\beta_{2}=1000$$

(15) E/R=5400

(16) $$\beta_{3}=7500$$

The cement hydration model is valid for concrete with various mixing proportions (ordinary-strength concrete and high-strength concrete), various curing temperature histories, and different cement types. The input parameters of the hydration model are concrete mixing proportions, cement compound compositions, Blaine surface area, and concrete curing temperatures. By using input parameters, the values of coefficients of the hydration model can be determined by Equations (9)–(16). Furthermore, the time-dependent degree of hydration can be calculated by Equation (1).

### 2.2. Dilution Effect, Nucleation Effect, and Chemical Effect of Limestone Particles

Limestone powder presents a dilution effect, physical effect (nucleation effect), and chemical effect (formation of monocarboaluminate phase) on cement hydration [15].

Dilution effect: limestone replacement increases the effective W/C ratio and improves the hydration rate of cement. For high-strength concrete with low water-to-binder ratios, the effective W/C ratio can be significantly increased because of limestone replacement. This dilution effect is considered by using the $C_{0}/W_{0}$ item in Equation (4).

Physical effect (nucleation effect): Limestone particles can provide additional sites for the nucleation and growth of cement hydration products, which generally enhances the cement hydration [9,15]. Maekawa et al. [9] found that for cement-limestone blends, hydrates were similarly formed on the overall surfaces of both particles of cement and limestone powder. The outer layer of hydrates could be precipitated from the eluted ion phase at any location, even away from cement particles, and all surface areas of particles can contribute as a precipitation site. In this study, the ratio of surface area between limestone powder and cement is used as an indicator to express the acceleration effect of limestone powder on cement hydration. This indicator is expressed as follows:

(17) $$L_{r}=\frac{LS_{0}\times S_{LS}}{C_{0}\times S_{C}}$$

where $L_{r}$ is the limestone nucleation effect indicator, $LS_{0}$ is the mass of limestone in concrete mixing proportions, and $S_{LS}$ is the Blaine surface area of limestone powder.

Maekawa et al. [9] proposed that for cement-limestone blends, the reaction rate of cement in the reaction-controlling stage and the diffusion-controlling stage is improved by increasing the limestone content. For the cement hydration model shown in Equation (1), the reaction-controlling stage relates to the reaction coefficient, $k_{r}$, and the diffusion-controlling stage relates to the diffusion coefficient, $D_{e}$. In this study, we assumed that $k_{r}$ and $D_{e}$ increase linearly with the limestone nucleation effect indicator, $L_{r}$. The relation between $k_{r}$ and $L_{r}$ is shown as follows:

(18) $$k_{rLS}=k_{r}(1+A_{1}L_{r})$$

where $k_{rLS}$ is the updated reaction coefficient, $k_{r}$, and $A_{1}$ is the enhanced coefficient of $k_{r}$.

Similarly, the relation between $D_{e}$ and $L_{r}$ is shown as follows:

(19) $$D_{eLS}=D_{e}(1+A_{2}L_{r})$$

where $D_{eLS}$ is the updated reaction coefficient, $D_{e}$ and $A_{2}$ is an enhanced coefficient of $D_{e}$. Through the analysis shown later, the values of $A_{1}$ and $A_{2}$ are set as the same values in 1.12. In addition, it should be noted that Equation (19) is a phenomenon-based equation used to describe the increase in the rate of hydration in the diffusion-controlled stage of PLC concrete. Equation (19) does not accurately analyze the relationship between parameter $D_{eLS}$ and the porosity of calcium silicate hydrate (C–S–H).

Chemical effect: Aside from the dilution effect and nucleation effect, limestone particles can react slightly with the cement and form a mainly monocarboaluminate phase. The chemical reactivity of limestone is very weak compared to cement. Ye et al. [11,24] proposed that limestone can be approximately regarded as a chemically inert material. Hence, the chemical effect of limestone powder is not considered in this study. The chemical reactivity of limestone [15] should be examined in further studies. In addition, it is assumed that the activation energy of the various chemical processes does not change with limestone replacement [9].

The proposed blended-hydration model considers the enhancing effects from limestone replacements. The dilution effect and physical effect (nucleation effect) of limestone are modeled. The dilution effect is considered through capillary water concentration (Equation (4)). The physical effect (nucleation effect) is considered through a limestone nucleation effect indicator. Compared to the plain cement hydration model, the limestone-blended cement hydration model utilizes additional input parameters that represent the limestone content in concrete mixing proportions and the Blaine surface area of the limestone. The values of coefficients of the blended-cement hydration model can be determined by using input parameters (Equations (9)–(16), Equations (17)–(19)). Furthermore, the time-dependent degree of hydration in cement-limestone blends can be calculated (Equation (1)).

## 3. Gel–Space Ratio and Compressive Strength

It is well known that the compressive strength of concrete depends on the gel–space ratio determined from the degree of cement hydration and the W/C ratio [9]. The gel–space ratio is equal to the ratio of the volume of cement hydration products to the sum of the volume of reacted cement and capillary pores. For Portland cement, 1 mL of hydrated cement occupies approximately 2.06 mL of space. Therefore, the gel–space ratio, $x_{fc}$, [25] of concrete can be calculated as follows:

(20) $$x_{fc}=\frac{2.06(1/\rho_{C})\alpha C_{0}}{(1/\rho_{C})\alpha C_{0}+W_{0}}$$

Furthermore, the development of the compressive strength of concrete can be evaluated by Powers’ strength theory [9,25] as follows:

(21) $$f_{c}=\sigma_{0}{x_{fc}}^{n}$$

where $f_{c}$ is the compressive strength of concrete, $\sigma_{0}$ is the intrinsic strength of the material, and n is the strength exponent. For PLC concrete, the degree of hydration can be calculated by using the kinetic hydration model (shown in Section 2). Furthermore, the gel–space ratio and compressive strength can be calculated based on the degree of hydration.

## 4. Carbonation Model of Concrete

Carbonation is related to the material properties and environmental conditions of concrete. The amount of carbonatable materials in concrete, such as calcium hydroxide (CH) and C-S-H, is dependent on cement and the supplementary cementing material content in concrete mixing proportions and the reaction degree of binders. For plain concrete or for concrete that incorporates chemically inert materials, CH contents can be determined as follows:

(22) $$CH(t)=RCH_{CE}\times C_{0}\times \alpha$$

where $RCH_{CE}$ is the mass of CH from the hydration of 1 unit mass of cement. $RCH_{CE}$ can be determined by using the mineral compositions of cement [26,27].

Similarly, by using cement content and the reaction degree of cement, C-S-H contents can be calculated as follows:

(23) $$CSH(t)=2.85f_{S,C}\times C_{0}\times \alpha$$

where $f_{S,C}$ is the silica weight fraction in cement. The 2.85 coefficient represents the mass ratio between C-S-H molar weight and SiO_2_ weight in C-S-H [26,27].

The hydration products from cement hydration deposit in the capillary pore spaces of concrete, and the porosity of concrete will reduce because of cement hydration. The porosity of concrete, *ε*, can be determined as follows:

(24) ε(t)=W0ρW−0.25×C0×α−ΔεC

where $\Delta \varepsilon_{C}$ is the porosity reduction due to the carbonation of concrete [26,27].

Demis et al. [17] proposed that when relative humidity in the environment is higher than 0.55, the carbonation of concrete is controlled by the diffusion of CO_2_. The carbonation depth of concrete can be determined as follows [17]:

(25) $$x_{c}=\sqrt{\frac{2D{\left[CO_{2}\right]}_{0}t}{\left[\mathrm{CH}]+3[\mathrm{CSH}\right]}}$$

(26) $$D=A{\left(\frac{\varepsilon }{\frac{C_{0}}{\rho_{c}}+\frac{W_{0}}{\rho_{w}}}\right)}^{a}{\left(1-\frac{RH}{100}\right)}^{2.2}$$

where $x_{c}$ is the carbonation depth of concrete, D is CO_2_ diffusivity, ${\left[CO_{2}\right]}_{0}$ is CO_2_ molar concentration at the concrete surface, [CH] is molar concentration of calcium hydroxide, [CSH] is molar concentration of calcium silicate hydrate, *A* and *a* are carbonation reaction parameters, and RH is the environmental relative humidity. [CH]+3[CSH] in the denominator of Equation (26) is the content of carbonatable material [26,27]. The dependence of CO_2_ diffusivity on temperature can be considered by using Arrhenius’s Law [28,29,30].

**Figure 1:**
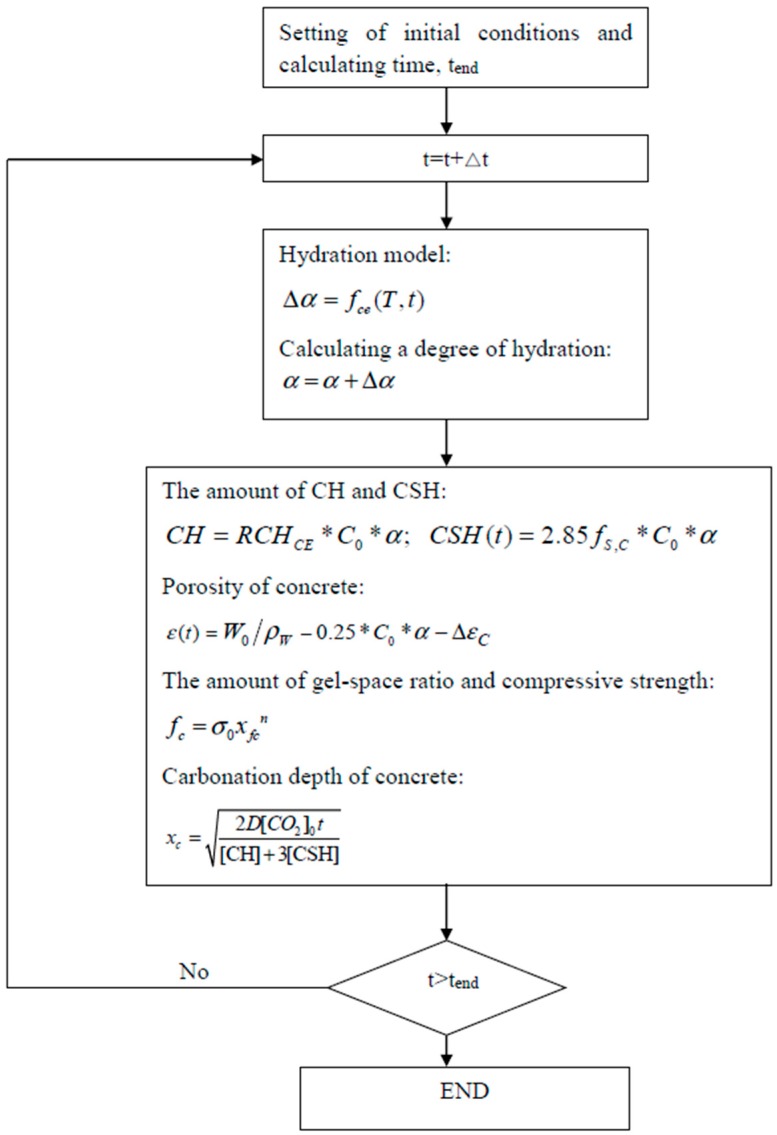
Flowchart of the model.

The flowchart of the proposed model is shown in Figure 1. By using the hydration model, the hydration degree of cement is calculated. The influences of concrete mixing proportions, binder properties, and curing conditions on the rate of hydration are considered. Furthermore, the carbonatable material contents, porosity, gel–space ratio, and the compressive strength of concrete are calculated by using the degree of hydration and the concrete mixing proportions. Finally, the carbonation depth is evaluated by considering the concrete materials’ properties and environmental conditions.

## 5. Verification of the Proposed Model

### 5.1. Degree of Cement Hydration in Cement-Limestone Blends

**Figure 2:**
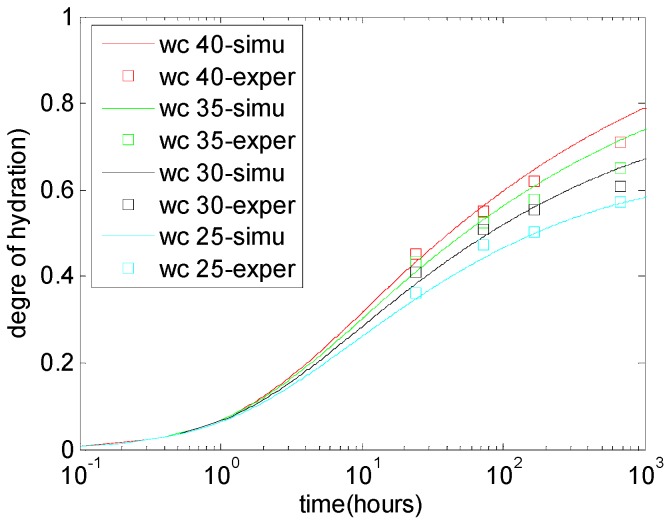
Degree of hydration in plain cement paste [3].

The experimental results from Bonavetti et al. [3] were used to verify the hydration model for limestone-blended cement. Bonavetti et al. [3] measured the degree of cement hydration in cement-limestone blended paste. The water-to-binder ratios of paste specimens ranged from 0.25–0.4. The limestone powder replaces cement at two levels: 9% and 18%. The specimens were stored in a water bath at 21 °C until the test ages. At ages of 1, 3, 7, and 28 days, the degree of hydration was estimated by the non-evaporable water content. By using the cement hydration model, the degree of hydration in plain cement paste was calculated and is shown in Figure 2. When the W/C ratio increases, the available depositing space for cement hydration products increases, the concentration of capillary water increases, and the rate of cement hydration and the degree of hydration also increase.

**Figure 3:**
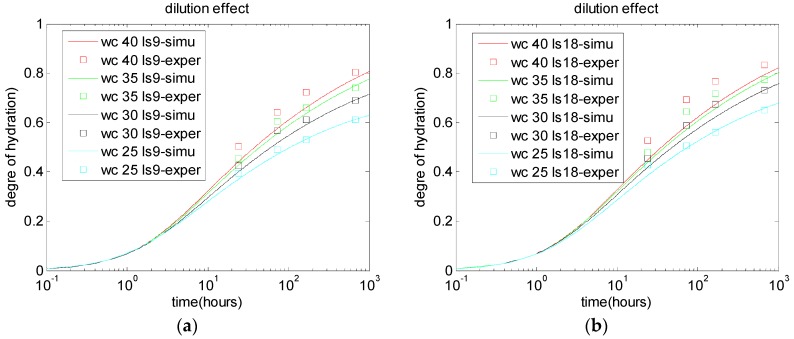
Dilution effect of limestone replacements: (**a**) 9% limestone powder; and (**b**) 18% limestone powder [3].

For cement-limestone blended paste, because of the limestone replacement, the effective W/C ratio increases. Consequently, the degree of cement hydration in cement-limestone blends is higher than that in control paste. This dilution effect is considered by using the $C_{0}/W_{0}$ item in Equation (4). By using the cement hydration model, the limestone dilution effect is calculated, and is shown in Figure 3. With increasing limestone replacement ratios, the improvements regarding the degree of cement hydration become more significant. However, the calculated results for degree of hydration are slightly lower than the experimental results, because the nucleation effect was ignored.

**Figure 4:**
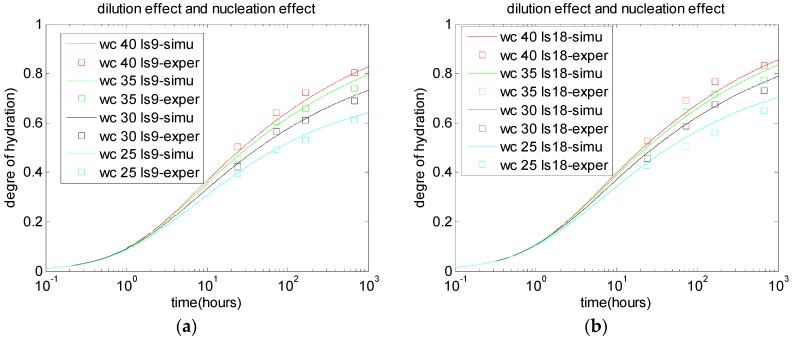
Dilution effect and nucleation effect of limestone replacements: (**a**) 9% limestone powder; and (**b**) 18% limestone powder [3].

In this study, the limestone nucleation effect indicator, $L_{r}$, is proposed to consider the physical effect of limestone powder. By using the updated reaction coefficients, $k_{r}$ and $D_{e}$, the physical effect of limestone powder can be taken into account. The enhanced coefficients $A_{1}$ and $A_{2}$ can be calibrated based on the experimental results for the degree of hydration. The values of $A_{1}$ and $A_{2}$ are set as 1.12. As shown in Figure 4, when the dilution effect and nucleation effect are considered, the analyzed results show better agreement with experimental results than those shown in Figure 3.

In this study, experimental results [3] are only considered starting from one day of hydration. The isothermal calorimeter is widely used to study the very early age hydration behavior for aging of less than one day [31,32,33,34,35,36]. By using an isothermal calorimeter, Aqel et al. [35] found that for concrete with a water-to-binder ratio of 0.37 and a limestone replacement ratio of 10%, after 20 h, the heat of hydration increased by approximately 5% compared with control concrete. Bouasker et al. [31] found that for concrete with a water-to-binder ratio of 0.32 and a limestone replacement ratio 10%, after one day the heat of hydration decreased by approximately 17% as compared to control concrete. In this study, for concrete with a water-to-binder ratio of 0.37 and a limestone replacement ratio of 10%, after one day, the relative degree of hydration is approximately 1.1. Hence, the heat of hydration almost did not change (hydration heat = mass of cement × hydration degree = (1 − 10%) × 1.1 = 0.99). The differences between the studies of Aqel et al. [35] and Bouasker et al. [31], and this study may come from the different crystal structures of limestone [36] or the calibration of $A_{1}$. The calibration of $A_{1}$ requires further study by using actual measurements in very early aged concrete.

### 5.2. Compressive Strength of Concrete

**Figure 5:**
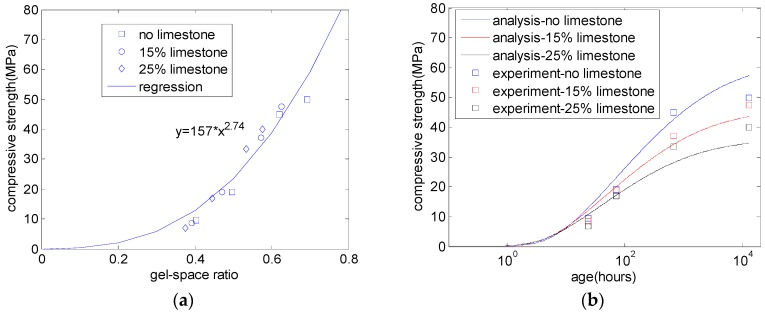
Compressive strength of PLC concrete: (**a**) the relation between strength and gel–space ratio; and (**b**) the predicted compressive strength [6].

Experimental results for the compressive strength of PLC concrete from Parrot [6] were used to verify the compressive strength model. The water-to-binder ratio was 0.59, and the binder content was 320 kg/m^3^. Limestone replaced cement at two levels: 15% and 25%. The concrete specimens were sealed and cured at 20 °C. At the ages of one day, three days, 28 days, and 18 months, the compressive strength of concrete was measured. The ages of one day and three days represent the early ages of concrete, and the age of 18 months represents the long-term age of concrete. Using the hydration model that considers the limestone dilution effect and the physical effect, the reaction degree of cement, and the gel–space ratio of concrete were calculated. Furthermore, the coefficients of $\sigma_{0}$ and n were set as 157 and 2.74, respectively, based on the experimental results [6]. Bentz et al. [5] proposed that the strength exponent, n, was between 2 and 3. The calibrated value of n in this study generally agrees with that value. As shown in Figure 5, the calculation results generally agree with the experimental results. However, the predicted long-term compressive strength deviates from the experimental results. This is because the formation of a monocarboaluminate phase at late ages was not considered in the proposed model.

**Figure 6:**
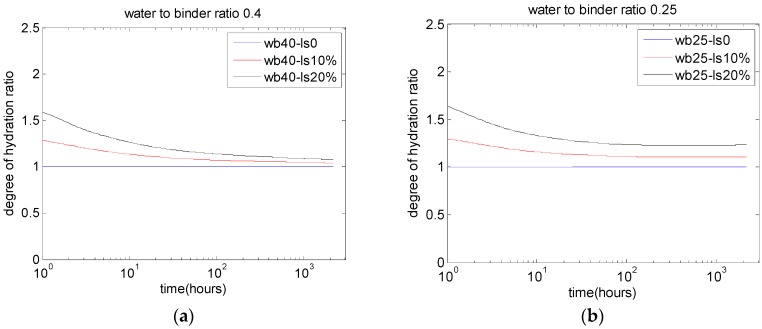
Effects of limestone replacements on the ratio of degree of hydration: (**a**) water-to-binder ratio of 0.4; (**b**) water-to-binder ratio of 0.25.

**Figure 7:**
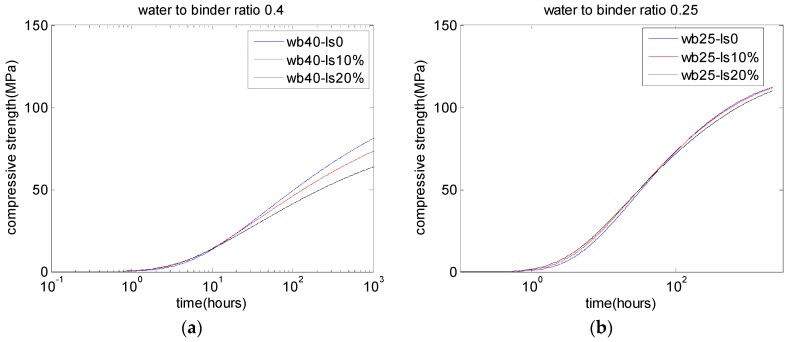
Effects of limestone replacements on compressive strength development: (**a**) water-to-binder ratio of 0.4; (**b**) water-to-binder ratio of 0.25.

**Figure 8:**
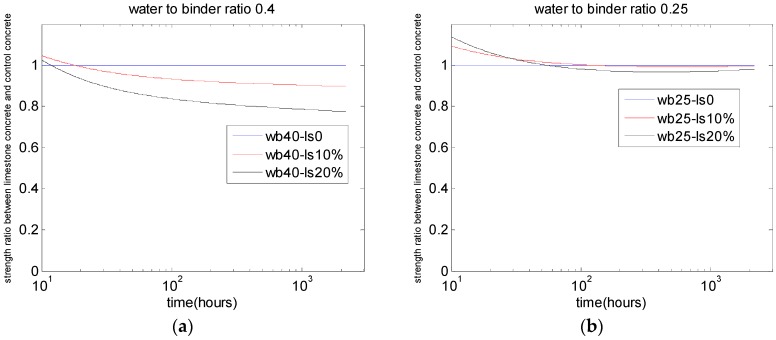
Effects of limestone replacements on the compressive strength ratio: (**a**) water-to-binder ratio of 0.4; (**b**) water-to-binder ratio of 0.25.

Figure 6, Figure 7 and Figure 8 show the parameter analysis of the interactions between water-to-binder ratio, limestone replacement ratio, and strength development.

Figure 6 shows the analysis results of the ratio of the degree of hydration between PLC concrete and control concrete. The results show that the limestone replacement can increase the degree of cement hydration. For concrete with a lower water-to-binder ratio (Figure 6b, water to binder ratio 0.25), when cement is partially replaced with limestone, the W/C ratio significantly increases. Consequently, the ultimate degree of cement hydration is significantly improved. Bonavetti et al. [3] also found that the limestone enhancement effect for the degree of hydration is significant for concrete with low water-to-binder ratios (shown in Figure 2 and Figure 4).

Figure 7 shows the analysis results for the compressive strength development of concrete containing various limestone contents. The results show that the limestone replacement can increase the early-age strength of concrete. At late ages, for concrete with higher water-to-binder ratio (Figure 7a, water-to-binder ratio is 0.4), the compressive strength of PLC concrete is lower than that of control concrete. On the other hand, for concrete with lower water-to-binder ratios (Figure 7b, water-to-binder ratio is 0.25), the compressive strength of PLC concrete is similar to that of control concrete. Bentz [15] also found that the impairment of compressive strength due to limestone replacements is not significant for concrete with low water-to-binder ratios.

Limestone presents positive and negative effects on the strength of concrete. Limestone can increase the degree of hydration of cement, which will increase the compressive strength of concrete. However, when cement is partially replaced by limestone, the amount of cement is reduced. This will decrease the compressive strength of concrete. Therefore, the compressive strength of PLC concrete is affected by both increasing and decreasing factors. Hence, the results of compressive strength are different from those of degree of hydration.

Figure 8 shows the analysis results for the ratio of compressive strength between PLC concrete and control concrete. The use of limestone powder in concrete with lower water-to-binder ratios is a more rational option, considering the compressive strength of concrete (Figure 8b, water-to-binder ratio is 0.25).

In this study, compressive strength development is evaluated by using gel–space ratio and Powers’ strength theory. However, the compressive strength model shows some limitations. First, Powers’ strength theory assumes that compressive strength development starts when cement hydration begins. While Carette et al. [33,34] reported that there is a threshold value for the degree of hydration. When the degree of cement hydration is lower than this threshold value, the compressive strength of concrete is zero. When the degree of cement hydration is higher than this threshold value, the development of compressive strength starts. The threshold for degree of hydration is not considered in this study. Second, the monocarboaluminate phase is produced from the chemical reaction of limestone [32]. The contribution of monocarboaluminate phase on strength development is not considered.

### 5.3. Carbonation of PLC Concrete

**Figure 9:**
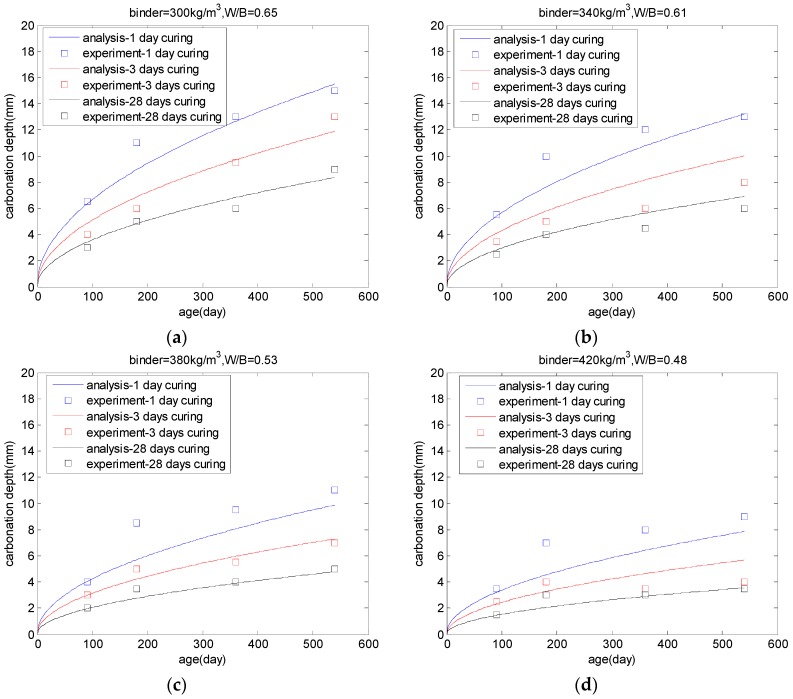
Carbonation depth of PLC concrete. The water-to-binder ratio (**a**) 0.65; (**b**) 0.61; (**c**) 0.53; and (**d**) 0.48 [7].

The experimental results for the carbonation of PLC concrete from references [6,7] were used to verify the proposed carbonation model. Balayssac et al. [7] investigated the carbonation resistance of PLC concrete with different binder contents and curing periods. The concrete mixing proportions are shown in Table 1. The limestone replacement ratio was 0.25. The concrete samples were wet cured at 20 °C before being exposed to a natural carbonation environment. After one day, three days, and 28 days of wet curing, the concrete was exposed to a natural carbonation environment (20 °C and 60% relative humidity). After 90, 180, 360, and 540 days of exposure, the carbonation depths were measured after application of phenolphthalein to the concrete-free surface. The carbonation depths of the concrete specimens were calculated by using Equation (25) and are shown in Figure 9 (*A* = 1.1 × 10^−5^ and *a* = 4.7). With increasing water-to-binder ratios or decreasing cure periods, the carbonation depth of concrete increases. When the curing period increases from one day to three days, the concrete carbonation depth decreases by approximately 25%. While the curing period increases from three days to 28 days, the concrete carbonation depth decreases by approximately 25%–30%. This is because the rate of cement hydration at early ages (before three days) is much quicker than that at late ages (28 days). Hence, early age curing is effective for reducing the carbonation depth of concrete.

**Figure 10:**
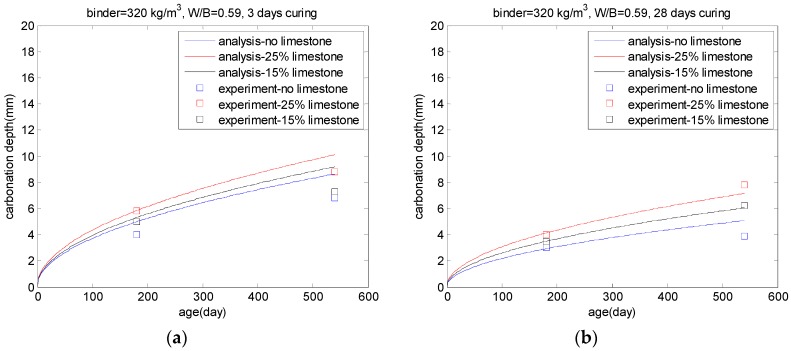
Effects of limestone replacements on carbonation: (**a**) three-day cure before carbonation; (**b**) 28-day cure before carbonation.

Parrot [6] investigated the effect of limestone replacements on carbonation. Control concrete and PLC concrete (limestone replacement ratios 0.15 and 0.25) were employed. After three or 28 days of curing, the concrete specimens were exposed to a natural carbonation environment (20 ℃ and 60% relative humidity). After 6 months and 18 months of exposure, carbonation depths were measured. Figure 10 shows the effect of limestone replacement on carbonation. Compared with control concrete, PLC concrete shows a higher carbonation depth. For three-day curing concrete incorporating 25% limestone, after 18 months carbonation, the carbonation depth is about 15% higher than that of control concrete (shown in Figure 10a). For the same concrete after 28 days of curing, the carbonation depth of concrete is approximately 30% higher than that of control concrete (shown in Figure 10b). This can be explained through the ratio of the degree of hydration (shown in Figure 6). The enhancement of the degree of hydration from limestone replacements is much more obvious in the early aging of concrete.

The carbonation resistance of PLC concrete depends on a trade-off between some competing effects. First, the limestone replacement increases the degree of cement hydration. This factor increases the carbonation resistance of PLC concrete. Second, limestone replacement reduces the cement content in concrete. This factor decreases the carbonation resistance of PLC concrete. The increasing and deceasing factors contribute to the carbonation resistance of concrete with different weights. The carbonation resistance of PLC concrete relates to increasing and deceasing factors.

In this study, the effects of carbonatable material contents, porosity, and environmental conditions on concrete carbonation are considered. However, the carbonation model also shows some limitations. First, the drying of concrete during carbonation tests was not considered [9]. The dependence of CO_2_ diffusivity on local relative humidity requires additional examination. Second, the change in carbonation depth may also be related to the changes in the pore solution and the composition of the cement hydrates with limestone present (and reacting) in the cement paste. This point was not considered.

## 6. Conclusions

This study presents a systematic study on hydration, strength development, and carbonation of PLC concrete. The conclusions of this study are summarized as follows:

First, a kinetic hydration model is proposed for cement-limestone blends. The dilution effect and physical effect from limestone powder replacements are taken in account. The dilution effect is considered by the effective W/C ratio. The physical effect is considered by the limestone nucleation effect indicator. The input parameters of the hydration model are concrete mixing proportions, compound compositions, Blaine surface area of cement and limestone, and concrete curing temperatures. The values of the reaction coefficients of the hydration model can be determined using the input parameters. Furthermore, the time-dependent degree of hydration can be calculated.

Second, the compressive strength of PLC concrete is evaluated using the gel–space ratio. Limestone replacement can increase the degree of cement hydration. For concrete with low water-to-binder ratios, the improvements related to the degree of cement hydration are more obvious. At late ages, for concrete with higher water-to-binder ratios, limestone replacements impair compressive strength, while, for concrete with lower water-to-binder ratios, strength reduction due to limestone replacements is marginal. Regarding compressive strength, the use of limestone in concrete with low water-to-binder ratios is a more rational option.

Third, the calculation results from the hydration model, such as carbonatable material contents and porosity of concrete, are used as input parameters for the carbonation model. CO_2_ diffusivity and carbonation depth of PLC concrete are evaluated by considering concrete material properties and environmental conditions. With increasing water-to-binder ratios and limestone content or reductions in curing period, the carbonation depth of concrete increases. The carbonation resistance of PLC concrete is related to both increasing factors (limestone replacements can increase the reaction degree of cement) and deceasing factors (limestone replacements decrease cement contents in mixing proportions).

## Figures and Tables

**Table 1:** Mixing proportions of concrete.

Binder (kg/m^3^)	Water-to-Binder Ratio	Gravel/Sand	28 Days Compressive Strength (MPa)
300	0.65	1	25.1
340	0.61	1.13	32.6
380	0.53	1.13	37.8
420	0.48	1.15	43.5
